# Complete Genome Sequence of Lactococcus lactis subsp. cremoris 3107, Host for the Model Lactococcal P335 Bacteriophage TP901-1

**DOI:** 10.1128/MRA.01635-18

**Published:** 2019-01-17

**Authors:** Andrea Erazo Garzon, Jennifer Mahony, Francesca Bottacini, Philip Kelleher, Douwe van Sinderen

**Affiliations:** a School of Microbiology, University College Cork, Cork, Ireland; b APC Microbiome Ireland, University College Cork, Cork, Ireland; University of Delaware

## Abstract

The complete genome sequence of Lactococcus lactis subsp. cremoris 3107, a dairy starter strain and a host for the model lactococcal P335 bacteriophage TP901-1, is reported here. The circular chromosome of L. lactis subsp. cremoris 3107 is among the smallest genomes of currently sequenced lactococcal strains. L. lactis subsp. cremoris 3107 harbors a complement of six plasmids, which appears to be a reflection of its adaptation to the nutrient-rich dairy environment.

## Abstract (precis)

The complete genome sequence of Lactococcus lactis subsp. cremoris 3107, a dairy starter strain and a host for the model lactococcal P335 bacteriophage TP901-1, is reported here.

## ANNOUNCEMENT

The dairy fermentation industry relies on starter or adjunct cultures, predominantly composed of lactic acid bacteria (LAB), to produce high-quality end products (1). Like all bacteria, LAB strains are susceptible to infection by bacteriophages (phages), which are ubiquitous in the dairy environment and largely insensitive to pasteurization treatments (2). A major economic concern for the dairy fermentation industry is phage attack of starter and adjunct strains during the fermentation process, which may negatively impact the final product quality and production regimes; this may thus lead to significant economic losses (3). Among the LAB, strains of the industrially significant species Lactococcus lactis are particularly susceptible to infection by phages (4, 5). Lactococcus lactis subsp. cremoris 3107 is the host of the model lysogenic lactococcal P335 phage TP901-1 (6). Because this L. lactis strain is host to an important model phage, its genome was sequenced to better characterize genes involved in phage-host interactions. The genome consists of a single 2.4-Mb chromosome (36% GC content) and six plasmids ranging in size from 2 to 60 kb, and analysis of the genes carried on both the chromosome and plasmids suggests that the plasmids are required for metabolism in the nutrient-rich dairy environment.

For PacBio sequencing, L. lactis subsp. cremoris 3107 cell pellets (from an overnight culture; see below) containing 10^9^ CFU were provided to GATC Biotech Ltd. (Germany) to perform chromosomal DNA extraction, library construction, and single-molecule real-time (SMRT) sequencing on Pacific Biosciences RS (run 1) and RS II (run 2) sequencing platforms. The library for PacBio sequencing was prepared using the SMRTbell template prep kit with 8- to 12-kb inserts, according to the manufacturer’s instructions (Pacific Biosciences, Menlo Park, CA, USA). *De novo* genome assembly of the SMRT sequencing data was performed using the RS_HGAP_Assembly.2 protocol (default parameters) implemented in the Pacific Biosciences SMRT Analysis portal (version 2.3.1). Quality filtering was performed automatically during assembly using the SMRT Portal P-filter module. Two SMRT cells were used for PacBio sequencing to achieve an initial assembly of the derived 52,984 filtered reads into 23 contigs, with an *N*_50_ contig length of 337,497 bp and an average reference coverage of 60.37×.

For Illumina-based sequencing, 5 µg chromosomal DNA from L. lactis subsp. cremoris 3107 was extracted using phenol-chloroform-based extractions, as previously described (7), following overnight growth of the strain at 30°C in M17 broth (Oxoid, UK) supplemented with 0.5% (vol/vol) glucose. Genomic libraries were constructed using the TruSeq DNA PCR-free LT kit (Illumina) and 2.5 μg of genomic DNA, which was fragmented with a Bioruptor next-generation sequencing (NGS) ultrasonicator (Diagenode, USA), followed by size evaluation using the TapeStation 2200 system (Agilent Technologies). Library samples were loaded into a flow cell version 3 (600 cycles; Illumina), and draft genome sequencing was performed on a MiSeq genomic platform (Illumina, UK) at GenProbio srl (Parma, Italy). Fastq files of the paired-end reads obtained from the genome sequencing were used as input for genome assemblies through the MEGAnnotator pipeline (8). The MIRA program (version 4.0.2) was used for *de novo* assembly of the genome sequence (9). The Illumina data were mapped on corresponding PacBio scaffolds to provide confidence in the generated sequence quality and to resolve base conflicts using Bowtie2 version 2.2.7, achieving a mapping coverage of 847× for the chromosome and an average coverage of 4,776× for the plasmids. Remaining low-quality regions or sequencing conflicts were resolved by primer walking and Sanger sequencing of PCR products (Eurofins MWG Operon, Germany).

Putative protein-encoding genes were identified using the prediction software Prodigal version 2.0 (10). Protein-encoding genes were automatically annotated using BLASTP version 2.2.26 (E value cutoff, 0.0001) sequence alignments against the nonredundant (nr) protein database curated by the NCBI (ftp://ftp.ncbi.nih.gov/blast/db/). Following automatic annotation, the obtained open reading frames (ORFs) were manually inspected and refined using the genome browser and annotation tool Artemis version 16 (11). Finally, ORF annotations were refined further where necessary using alternative functional searches using the PFAM database (12) and the Clusters of Orthologous Groups (COG) database (13). Predicted tRNA and rRNA genes were identified using tRNA-scan-SE version 1.4 (http://lowelab.ucsc.edu/tRNAscan-SE/) and RNAmmer version 1.2 (http://www.cbs.dtu.dk/services/RNAmmer/), respectively. Using Artemis version 16, the predicted RNA-specifying loci were manually added to the genome.

The complete genome content of L. lactis subsp. cremoris 3107 is represented by a single circular chromosome plus six plasmids (Table 1). The L. lactis subsp. cremoris 3107 genome is predicted to contain 2,380 protein-encoding genes, of which 101 are pseudogenes. The genome of L. lactis subsp. cremoris 3107 contains 164 transposase-encoding genes, including 20 copies of IS*712H* and 31 copies of IS*982B*. The high number of transposons and pseudogenes within this relatively small lactococcal chromosome suggests that the L. lactis subsp. cremoris 3107 genome has undergone significant genome decay while adapting to its environment. The L. lactis subsp. cremoris 3107 plasmids encode various traits for adaptation to the nutrient-rich dairy environment, such as lactose metabolism, making this strain suitable as a starter or adjunct culture.

**TABLE 1:** Genome features of *Lactococcus lactis* subsp. *cremoris* 3107

L. lactis subsp. *cremoris* 3107 chromosome or plasmid	Length (bp)	GC content (%)	Genome coverage (×)	
			PacBio	Illumina
Chromosome	2,378,982	35.82	65.01	846.88
p3107A	50,160	35.64	228.24	1,633.72
p3107B	60,216	33.38	230.17	1,550.52
p3107C	26,709	37.63	455.12	2,467.09
p3107D	2,232	33.56	615.77	11,381.27
p3107E	18,170	34.81	491.65	4,443.82
p3107F	4,199	31.6	332.51	7,180.93

### Data availability.

The complete chromosome and plasmids of L. lactis subsp. cremoris 3107 were deposited in GenBank under accession no. CP031538 (chromosome), CP031539 (p3107A), CP031540 (p3107B), CP031541 (p3107C), CP031542 (p3107D), CP031543 (p3107E), and CP031544 (p3107F). The SMRT and Illumina raw reads were deposited in SRA under BioProject no. PRJNA438435.
